# Enhanced Neurite Outgrowth of Human Model (NT2) Neurons by Small-Molecule Inhibitors of Rho/ROCK Signaling

**DOI:** 10.1371/journal.pone.0118536

**Published:** 2015-02-25

**Authors:** Frank Roloff, Hannah Scheiblich, Carola Dewitz, Silke Dempewolf, Michael Stern, Gerd Bicker

**Affiliations:** 1 Division of Cell Biology, University of Veterinary Medicine Hannover, Bischofsholer Damm 15/102, 30173, Hannover, Germany; 2 Center for Systems Neuroscience, Hannover, Germany; Goethe University Frankfurt, GERMANY

## Abstract

Axonal injury in the adult human central nervous system often results in loss of sensation and motor functions. Promoting regeneration of severed axons requires the inactivation of growth inhibitory influences from the tissue environment and stimulation of the neuron intrinsic growth potential. Especially glial cell derived factors, such as chondroitin sulfate proteoglycans, Nogo-A, myelin-associated glycoprotein, and myelin in general, prevent axon regeneration. Most of the glial growth inhibiting factors converge onto the Rho/ROCK signaling pathway in neurons. Although conditions in the injured nervous system are clearly different from those during neurite outgrowth in vitro, here we use a chemical approach to manipulate Rho/ROCK signalling with small-molecule agents to encourage neurite outgrowth in cell culture. The development of therapeutic treatments requires drug testing not only on neurons of experimental animals, but also on human neurons. Using human NT2 model neurons, we demonstrate that the pain reliever Ibuprofen decreases RhoA (Ras homolog gene family, member A GTPase) activation and promotes neurite growth. Inhibition of the downstream effector Rho kinase by the drug Y-27632 results in a strong increase in neurite outgrowth. Conversely, activation of the Rho pathway by lysophosphatidic acid results in growth cone collapse and eventually to neurite retraction. Finally, we show that blocking of Rho kinase, but not RhoA results in an increase in neurons bearing neurites. Due to its anti-inflammatory and neurite growth promoting action, the use of a pharmacological treatment of damaged neural tissue with Ibuprofen should be explored.

## Introduction

In general, the adult mammalian central nervous system (CNS) cannot regenerate injured axons. As a consequence, human patients with severe spinal cord injuries suffer from loss of motor control and sensation. The reasons for the inability to regenerate fall into two broad categories: the non-permissive tissue environment and neuron intrinsic factors [1]. Main obstacles in the neuronal environment are reactive astrocytes that generate chondroitin sulfate proteoglycans (CSPGs) forming scar tissue [2,3] and myelin-producing oligodendrocytes that expose myelin associated glycoprotein (MAG), Nogo-A [4] and oligodendrocyte myelin glycoprotein (OMgp) as growth-inhibitory factors [5–8] to the axons. These two broad classes of molecules are upregulated after neuronal injury and prevent regeneration beyond the lesion site. All of the mentioned extracellular growth inhibiting factors interact with various receptors on the axonal membrane and converge downstream on the small GTPase RhoA signaling pathway [8]. The activation of RhoA causes cytoskeletal changes eventually leading to a growth cone collapse which in turn suppresses axonal re-extension [9].

A rather promising approach for enabling axonal regeneration is the chemical manipulation of the Rho signaling cascade [8, 10–13]. In vitro and in vivo studies using rodents have shown that inhibition of Rho activation resulted in neurite outgrowth on non-permissive myelin and CSPG substrates and in improved sprouting of serotonin-positive fibers across the lesion site [11,13–16]. Moreover, blocking the downstream effector of RhoA, the Rho kinase (ROCK, Rho-associated coiled coil forming protein serine/threonine kinase) increased axonal regeneration in cultures of embryonic and adult rat neurons [11,17]. Non-steroid anti-inflammatory drugs (NSAIDs), such as ibuprofen do not only target cyclooxygenases, but suppress also Rho-A activation [18]. Translation of the neurite growth promoting effect of Rho manipulation into a therapeutic treatment of axonal damage requires testing of pharmaceutical agents not only in experimental animals, but also on human neurons. Several inhibitors of ROCK have been shown to partially restore neurite outgrowth of human (NT2, Ntera2, NT2/D1 precursor cells) neurons on non-permissive CSPG substrate [15].

Here, we investigated for the first time whether the analgetic Ibuprofen could enhance neurite outgrowth of human neurons on a permissive substrate. These model neurons were differentiated by retinoic acid treatment from the Ntera2/D1 clone of a human teratocarcinoma line [19] and have been well characterized in a variety of biomedical applications [20] including neurite outgrowth assays [15,21,22]. We asked whether a blocker (Y-27632, (1R,4r)-4-((R)-1-aminoethyl)-N-(pyridin-4-yl)cyclohexanecarboxamide) of ROCK activation, the other downstream therapeutic target, would affect neurite outgrowth with comparable efficacy. After treating the human model neurons with Ibuprofen, levels of RhoA activity were determined in a pull down assay. Since Rho/ROCK inhibition is known to change cytoskeletal dynamics, we compared the capability of the human neurons for neurite initiation under RhoA and ROCK inhibiting conditions.

## Materials and Methods

### Antibodies and reagents

Unless stated otherwise, all chemicals were obtained from Sigma-Aldrich (Taufkirchen, Germany). All test substances were diluted in Dulbecco´s modified eagle medium nutrient mixture F-12 (DMEM/F12, Gibco-Invitrogen, Karlsruhe, Germany) containing 10% fetal bovine serum (Gibco-Invitrogen), 1% Penicillin and Streptomycin (Gibco-Invitrogen) and 10 μM retinoic acid. The non-steroidal cyclooxygenase inhibitor Ibuprofen, the ROCK inhibitors Y-27632 and Fasudil, and the cAMP analogue 8-Br-cAMP (8-Bromoadenosine 3′, 5′-cyclic monophosphate) were purchased from Sigma-Aldrich. The RhoA Activation Assay Biochem Kit (bead pull-down format) was purchased from Cytoskeleton Inc. (Denver, CO, USA). Alamar Blue cell viability assay to measure cytotoxic effect was purchased from Trinova (Giessen, Germany).

### Neuronal differentiation

Human NT2/D1 precursor cells (NT2) were purchased from the American Type Culture Collection (ATTC, Manassas, VA, USA). Neuronal differentiation was carried out as previously described [21]. NT2 precursor cells were cultured in 95 mm bacteriological grade Petri dishes (Greiner, Hamburg, Germany). Cultures in each Petri dish had at least densities of 5 x 10^6^ cells/dish in 10 ml DMEM/F12 supplemented with 10% fetal bovine serum, 1% Penicillin and Streptomycin and 10 μM retinoic acid (RA medium) to start neuronal differentiation. Cells were cultured for 14 days in RA medium in a free floating sphere culture with medium change every 2–3 days. At the end of the incubation, cells were centrifuged, mechanically dispersed and counted. Dispersed cells were seeded to poly-D-lysine (10 μg/ml, Sigma-Aldrich) and Laminin (100 μg/ml, Sigma-Aldrich) coated 96-well-plates (Corning Costar, Kaiserslautern, Germany) at a density of 10,000 cells per well and with 8 wells per concentration. Experiments were performed with neurons treated for two weeks with retinoic acid (2wkRA) from passage 27 to 35.

### Neurite outgrowth assay

After cells had successfully attached to the plate, RA medium was changed against RA medium containing the test substances. Ibuprofen (10, 100, 500 μM) and 8-Br-cAMP (1 mM) were dissolved in RA medium. The ROCK inhibitor Y-27632 (1, 5, 10, 50 μM) was diluted to final concentration from a stock solution (10 mM) in H_2_O. The ROCK inhibitor Fasudil was diluted in culture media to final concentrations of 1 μM, 10 μM, and 100 μM. Cells were cultured for 24 hours under standard conditions (37°C, 5% CO_2_) in an incubator. Each experiment was performed in 8 wells per concentration/substance and was repeated at least 3 times. The next day, cells were washed with PBS (phosphate buffered saline) and subjected to the Alamar Blue cell viability assay to measure possible cytotoxic side effects.

### Cell viability assay / Toxicity assay

Survival of cells after treatment with Ibuprofen, Y-27632, Fasudil, and 8-Br-cAMP was quantified using the Alamar Blue cell viability assay. Cultures were incubated under standard conditions (37°C, 5% CO_2_) in 200 μl RA-medium and with 5% Alamar Blue for two hours. Alamar Blue fluorescence was measured for each well using a plate reader (Infinite M200, Tecan, Mainz, Germany) with an excitation wavelength of 560 nm and an emission wavelength of 590 nm at 37°C. Basal fluorescence of Alamar Blue solution was subtracted as blank from relative fluorescence values. Survival rate was calculated and standardized based on the means of control wells. For all used concentrations of the pharmacological agents, we found no significant decrease in viability.

### Immunocytochemistry

Stainings were performed as described earlier [21,22]. Cultures were rinsed with PBS to remove remaining Alamar Blue solution. Cells were than fixed with 4% PFA (paraformaldehyde) for 15 minutes at room temperature and washed 3 times in PBS containing 0.1% Triton X-100 (PBST) to remove remaining PFA. Unspecific protein binding sites were blocked with 5% normal horse serum in PBST for at least 60 minutes at room temperature. Monoclonal antibody β-III-Tubulin (1:10,000, Sigma) was applied overnight at 4°C. After 3 washing steps in PBST, neurons were incubated with biotinylated secondary antibody horse-against mouse (Vector, Burlingame, MA, USA) for 60 minutes at room temperature before 3 additional washing steps in PBST. Streptavidin coupled Cy3 was applied for 60 minutes at room temperature to detect immunofluorescence. To visualize nuclei we used DAPI (4′6-diamidino-2′henylindoldihydrochloride) at a concentration of 0.1 μg/ml as a counterstain.

### RhoA pull down assay

For detection of RhoA, we performed a RhoA pull down assay (Cytoskeleton, Denver, CO, USA). Lysates were collected from cells differentiated under control conditions or inhibition of RhoA and ROCK via Ibuprofen and Y-27632 respectively. Cells were cultured for 30 minutes (37°X, 5% CO_2_) on PDL and Laminin coated bacteriological 35 mm petri dishes (Falcon) at a density of 2x10^6^ cells. For lysate collection, cells were washed with ice cold PBS and then lysed in ice cold lysis puffer for 1 min. The lysate were centrifuged for 1 min at 4°C and 10,000g. The supernatants were snap frozen in liquid nitrogen and kept in a freezer until use in the pull down assay. Total protein quantification was performed with the Pierce BCA Protein Assay Kit (Thermo Scientific) and a plate reader (Infinite M200, Tecan). The RhoA pull down was performed according the manufacturer’s instructions. The cell lysates were incubated with Rhotekin-RBD beads at 4°C for at 60 min on a rocker. After centrifugation (5,000g for 1 min) at 4°C, the supernatant was nearly completely removed. After washing in wash buffer the remaining lysate was centrifuged again (5,000g for 1 min at 4°C).

### Western blotting

Prior to SDS-PAGE, 300 μg protein samples from the pull down assay were denatured at 95°C for 2 minutes in loading buffer (2× Laemmli buffer with 2% SDS, 10% 1 M Dithiothreitol). Samples were transferred to a precast 10% Tris-Glycine gel (NuSep, Wasserburg, Germany) and ran until the dye front reached the end of the gel. After equilibration of the gel in western blot buffer, the protein was transferred to a PVDF membrane. After blocking with 5% nonfat-dry milk in Tris Buffered Saline with Tween (TBST), RhoA was detected with the anti-RhoA (1:500 in TBST). Detection of the primary antibody was performed with a goat anti-mouse antibody (1:20,000) conjugated with biotin. The Vectastain ABC Kit (Vectorlabs, Peterborough, UK) was applied as recommended by the manufacturer for 30 minutes after 3 preceding wash steps with TBST. The bound biotin-avidin complex was visualized using the DAB peroxidase substrate kit (Vectorlabs). A brownish to black staining became visible after 10 minutes incubation. The reaction was stopped in distilled water. Using the open source tool ImageJ 1.47 (http://rsbweb.nih.gov/ij/), scanned blots were converted to inverted 8 bit grey scale images and analyzed with the ImageJ integrated gel analyze plugin. Grey values were normalized using the “sum of all data points in a replicate” according to Degasperi et al. 2014 [23].

### Microscopy and statistical analysis

Fluorescence images were taken with a Zeiss Axiovert 200 (Jena, Germany). The microscope was equipped with a CoolSnap camera (Photometrics, Tucson, AZ, USA) and MetaMorph software (Molecular Devices, Sunnyvale, CA, USA). Length of neurites was measured using the open source software of the NIH ImageJ 1.46d (Rasband W.S., ImageJ, U.S. National Institutes of Health, Bethesda, MD, http://rsb.info.nih.gov/jj/). The longest neurite of a neuron was measured from the soma to the tip. Additionally, the number of neurites for each neuron was counted and saved for later evaluation. Merging of channels, adjustment of contrast and brightness and addition of a scale bar were performed with ImageJ. Images were arranged with the GNU licensed layout tool Scribus 1.4.3. Statistical analysis was performed with Graph Pad Prism 5.0.1. Data are expressed as the mean ± SEM of at least 3 independent experiments. Data were analyzed according Gaussian distribution. If the data passed the normality test, significant differences were determined by ANOVA. Otherwise the data were analyzed with the Kruskal-Wallis-test with a Dunn´s post hoc test. Neurite outgrowth experiments were performed with 8 wells per concentration in at least 3 independent experiments. Significant levels are *<0.05, **<0.01, ***<0.001.

## Results

### RhoA/ROCK inhibition promotes neurite elongation of human neurons

**Fig 1:**
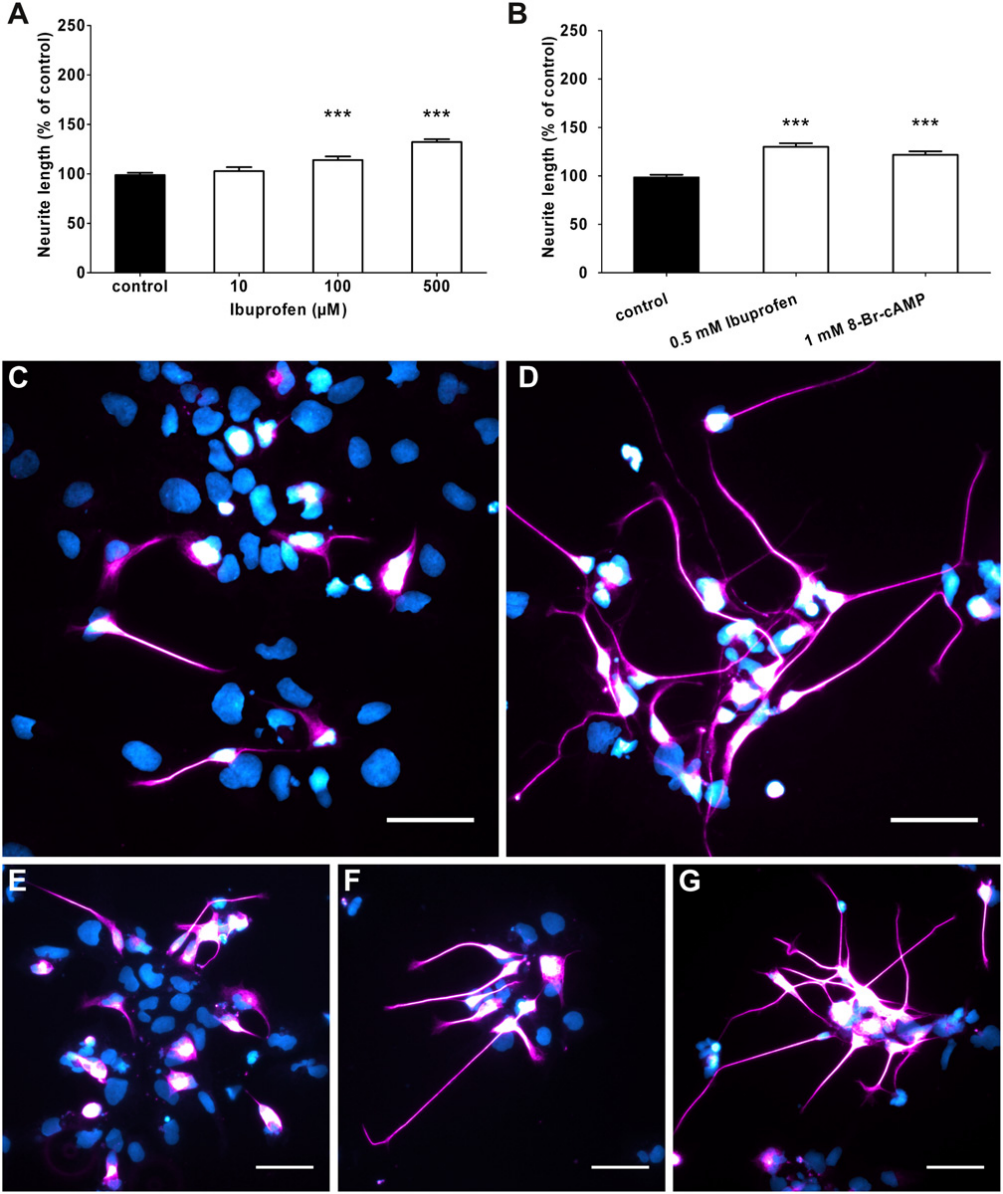
Ibuprofen increased neurite length of human model neurons. (A) Treatment with 100 μM and 500 μM Ibuprofen increased neurite outgrowth of human model neurons as measured after 24 hours. At 10 μM Ibuprofen treatment no difference to control could be detected. In 4 independent experiments, 778 to 2361 neurites were measured in total. (B) Neurite elongation under Ibuprofen treatment was slightly higher than in neurons treated with the membrane permeable analogue 8-Br-cAMP (5 experiments, 1015 to 2030 neurites measured. (C) Immunofluorescence staining of human model neurons under control conditions. (D) Neurons treated with 1 mM 8-Br-cAMP. (E-G) Neurons treated with 10 μM (E), 100 μM (F) and 500 μM (G) Ibuprofen. Cells are stained against beta-III-tubulin and counterstained with DAPI. ***p<0.001 with control by Kruskal-Wallis one-way ANOVA. Scale bars are 50 μm.

Culturing human NT2 2wkRA neurons with Ibuprofen resulted in an increased length of the longest neurite to 114.0% of control at 100 μM (Fig. 1A and F) and 132.2% of control at 500 μM (Fig. 1A and G). Lower concentrations (10 μM) failed to increase neurite length in a significant way (Fig. 1A and E). Moreover, we used a membrane permeable cAMP analogue to elevate intracellular levels of cAMP. Incubation with 1 mM 8-Br-cAMP resulted in a less, but still significant elongation of neurites to 121% of control (Fig. 1B and D).

**Fig 2:**
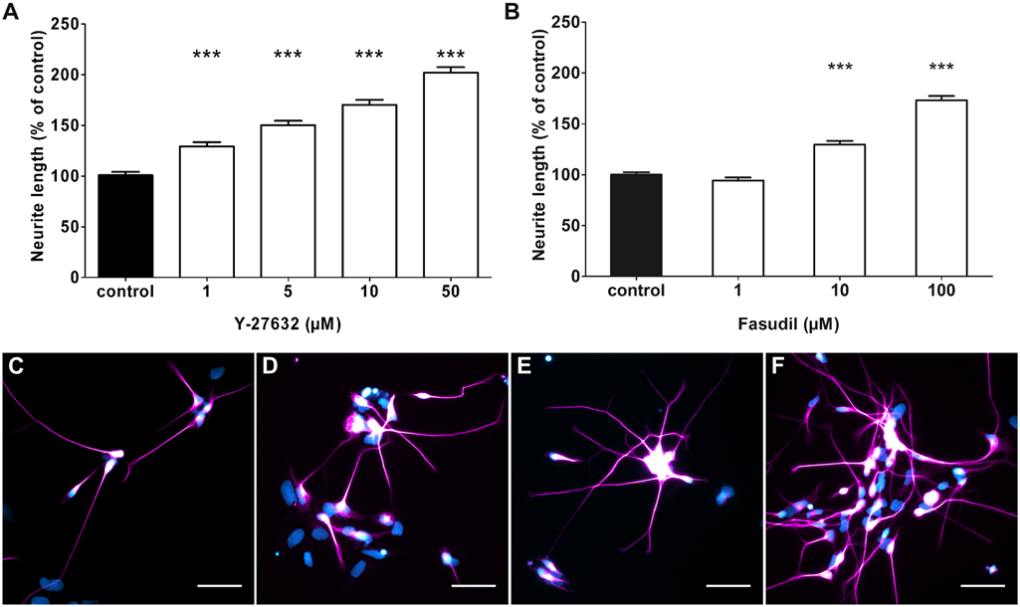
Rho kinase (ROCK) inhibitors promote neurite elongation of human model neurons. (A) Treatment with the ROCK inhibitor Y-27632 for 24 h resulted in dose-dependent increase of neurite lengths over a range from 1 μM to 50 μM Y-27632. At 50 μM Y-27632, treatment resulted in a doubling of neurite length compared to control conditions (3 experiments, 732 to 973 neurites measured). (B) Treatment with the ROCK inhibiting agent Fasudil led to increased neurite lengths of 125% of control at 10 μM and 175% at 100 μM Fasudil (3 experiments, 1511 to 2434 neurites measured) (C-F) Immunofluorescence staining of neurons treated with 1 μM (C), 5 μM (D), 10 μM (E), and 50 μM (F) of the ROCK inhibitor Y-27632. Neurons are stained against beta-III-tubulin and counterstained with DAPI. ***p<0.001 with control by Kruskal-Wallis one-way ANOVA. Scale bars are 50 μm.

Culturing the 2wkRA neurons with an inhibitor for the downstream effector of RhoA, the Rho Kinase resulted also in significantly longer neurites. Even low concentrations of 1 μM of the ROCK inhibitor Y-27632 caused an elongation to 129% of control (Fig. 2A), the level reached by application of 500 μM Ibuprofen (Fig. 1A and C). The dose dependent effect of Y-27632 showed an increased elongation to 150% of control at 5 μM (Fig. 2A and D), 170% of control at 10 μM (Fig. 2A and E), and a doubling to 202% of control at the highest Y-27632 concentration used (50 μM, Y-27632, Fig. 2A and F). To obtain independent support for the strategy of neurite growth enhancement by RhoA/ROCK inhibition, we used a second ROCK blocker. Treatment with the ROCK inhibitor Fasudil led to a similar increase in neurite lengths as seen for Y-27632 (Fig. 2B). The increase in neurite lengths was highly significant for Fasudil levels of 10 μM and 100 μM.

### Inhibition of RhoA/ROCK facilitates neurite initiation

**Fig 3:**
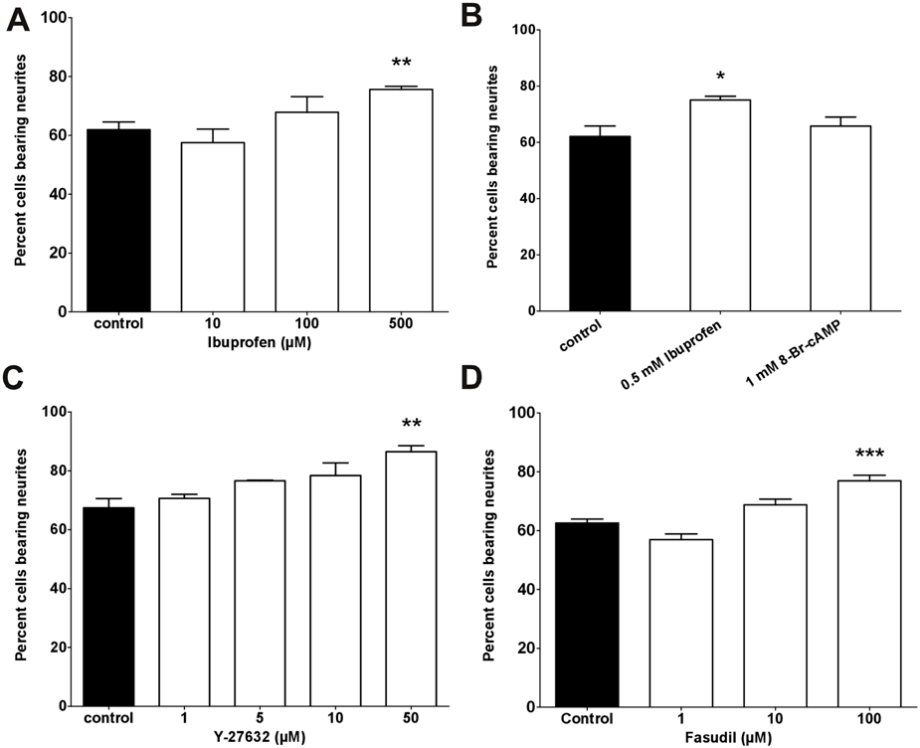
RhoA and Rho kinase inhibitors promote initiation of neurites. (A) The percentage of cells bearing a neurite was significantly increased at 500 μM of Ibuprofen whereas 10 μM and 100 μM led to no difference to control conditions (3–7 independent experiments, 744 to 2358 neurites measured). (B) Application of cAMP did not change the number of cells bearing a neurite, however neurite lengths were increased to a similar amount compared to Ibuprofen (5 independent experiments, 1151 to 1775 neurites measured). (C) Blocking Rho kinases with increasing levels of Y-27632 resulted in a trend to more cells bearing a neurite. A highly significant effect was observed at 50 μM (3 independent experiments, 732 to 973 neurites measured). (D) Blocking Rho kinases with elevated levels of Fasudil (100 μM) resulted in a highly significant increase in neurite bearing cells (3 independent experiments, 1857 to 3422 neurites measured). **p<0.01 and *p<0.05 against control by Kruskal-Wallis test.

In an attempt to test whether RhoA/ROCK inhibition directly effects elongation of existing neurites or whether the initiation of new neurites is altered, we categorized ß-tubulin III-positive cells into neurons with and without neurites. Under control conditions, approximately 60% of the neurons had grown at least one neurite (Fig. 3). Culturing neurons for 24 hours under Ibuprofen treatment resulted in a dose dependent increase from 57.52% at 10 μM, 67.85% at 100 μM, and to 75.62% at 500 μM Ibuprofen (Fig. 3A). However, only at the highest applied Ibuprofen concentration of 500 μM we obtained statistical significance. Culturing neurons under elevated levels of cAMP resulted in a weak but not significant increase of neurite bearing cells to 65.77% of ß-tubulin III-positive cells (Fig. 1B). Application of the ROCK inhibitor Y-27632 led to increased neurite formation shown as a dose dependent increase of neurite bearing cells (Fig. 3C). Under control conditions approximately 67% of all cells generated a neurite. After application of Y-27632, the percentage increased in a dose dependent manner to 86% at 50 μM Y-27632, which was significantly different from control. Application of the ROCK inhibitor Fasudil led to an increase in neurite bearing cells to approximately 75% at 100 μM (Fig. 3D).

### Activation of RhoA results in growth cone collapse and neurite retraction

**Fig 4:**
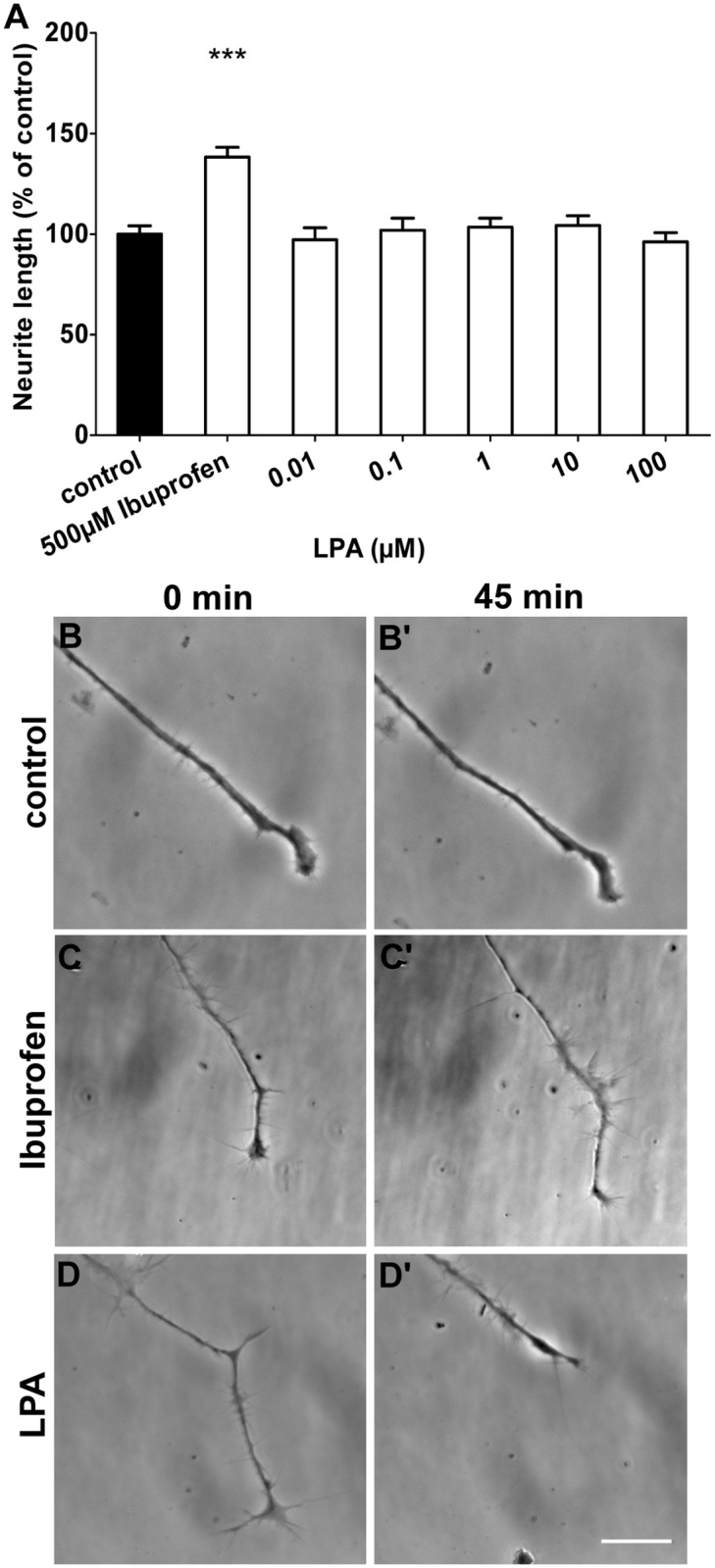
Rho activation resulted in a growth cone collapse, but not decreased neurite lengths. (A) Measurements of neurite outgrowth after 24 h incubation with elevated levels of the Rho activator lysophosphatidic acid (LPA) showed no change in neurite lengths, whereas Ibuprofen increased neurite length (2 independent experiments, 470 to 947 neurites measured). (B) Within 45 min, neurons cultured under control conditions showed a slowly advancing growth cone. (C) Inhibition of RhoA using 500 μM Ibuprofen elicited a weak positive effect on filopodial formation and extension of growth cone. (D) RhoA activation using 100 μM LPA resulted in a growth cone collapse and retraction of the neurite. ***p<0.001 against control by Kruskal-Wallis. Scale bar is 25 μm.

Inhibition of RhoA and Rho kinases resulted in enhanced neurite outgrowth compared to control conditions. We tested whether a potent RhoA activator, such as lysophosphatidic acid (LPA) had the opposite effect leading to decreased neurite lengths. After 24 hours incubation with LPA, neurite lengths did not differ from control (Fig. 4). Neurite lengths ranged from 103.5±4.5 percent of control at 1 μM LPA to 104.3±4.9 percent of control at 10 μM LPA. Neurons cultured with 500 μM Ibuprofen on the same cell culture plate showed a mean neurite length of 138.3±4.9 percent of control (Fig. 4).

We further checked whether RhoA activation could change shape and motility of growth cones on a shorter time scale. Culturing neurons with 10 μM LPA initially resulted in a broad growth cone collapse and neurite retraction within 45 minutes while growth cones under control conditions showed no reaction (Fig. 4B, D). On average, LPA administration caused a neurite retraction for about 65 μm over 45 minutes. Treatment with Ibuprofen induced no conspicuous effect on growth cone shape and behavior within this time interval (Fig. 4C).

**Fig 5:**
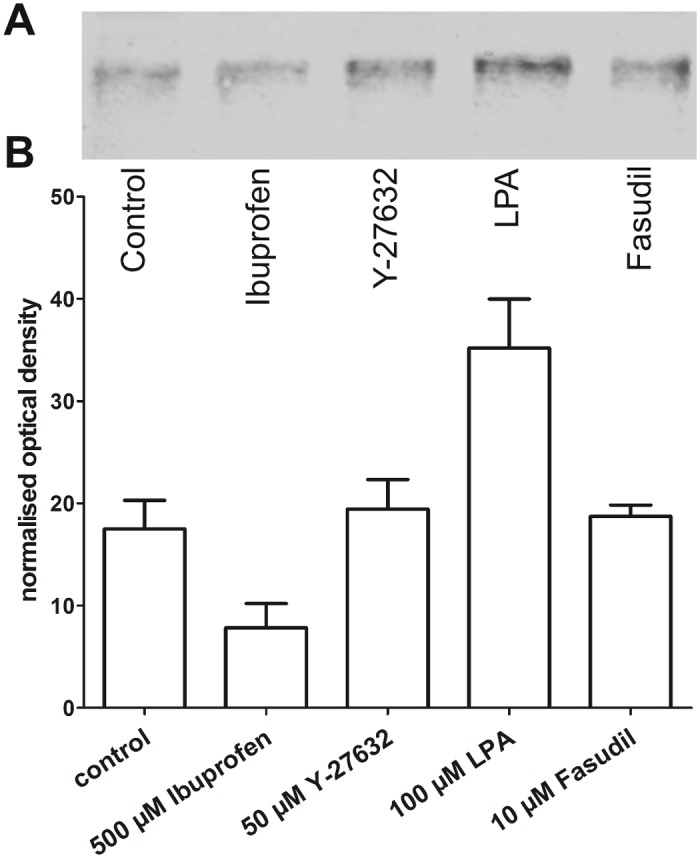
Pull down assay of RhoA. (A) A RhoA bead pull down assay revealed lowered levels of activated RhoA in Ibuprofen treated cells. Treatment with downstream Rho kinase inhibitor Y-27632 showed no difference in RhoA level compared to control. After treatment with lysophosphatidic acid, significantly more phosphorylated RhoA could be detected in the Western Blot. Treatment with downstream Rho kinase inhibitor Fasudil showed no difference in RhoA level compared to control. Cell lysates were blotted and stained with RhoA-specific monoclonal antibody. Staining is visualized with horseradish peroxidase and the 3, 3′-diaminobenzidine substrate. Molecular weight of stained protein bands corresponds to about 20 kDa as reported for RhoA. (B) Relative RhoA level was decreased to approximately 50% of control after Ibuprofen treatment whereas RhoA level after LPA treatment was increased to 200% of control. Data are normalized grey values ± SEM of 4 independent experiments.

Next, we measured the abundance of active RhoA in human model neurons under activating and inhibiting conditions in a pull down assay. Culturing neurons for 30 min with the Rho activator LPA caused an increased level of RhoA (Fig. 5). While the inhibitor Ibuprofen brought RhoA levels down to approximately 50% of control, incubation with the downstream Rho kinase inhibitor Y-27632 and Fasudil showed no effects on the level of activated RhoA.

## Discussion

Major obstacles for axonal regeneration after CNS injury are myelin-associated proteins, causing growth cone collapse and the formation of a glial scar, acting as physical barrier and containing growth-inhibitory proteoglycans [3]. For example, in spinal cord injury (SCI) the formation of the glial scar requires several weeks. This would enable at least a theoretical time window for axonal regeneration beyond the forming glial scar [24], if the growth-inhibitory environment of the myelin could be neutralized. Nevertheless, a remaining problem for functional recovery from CNS injuries is the long distance that some axons have to cover to reconnect to their synaptic targets. For developing therapeutic treatments, it would be necessary to provide an enhanced capability for outgrowth to the severed axons. Using human NT2 neurons in an in vitro assay, we have previously shown that the cAMP/PKA (protein kinase A, cAMP-dependent kinase) pathway [21] and co-culture with olfactory ensheathing cells [22] enhance the capability for neurite outgrowth. In this investigation, we focused on the Rho/ROCK pathway because it integrates many extracellular growth inhibitory signals of the CNS tissue [18], serving as a key regulator of the axonal growth state.

### Ibuprofen and Y-27632 enhance neurite length of human model neurons

In vitro treatment of human model neuron cultures with Ibuprofen increased neurite length to a similar extent as elevating cAMP levels (Fig. 1A, B). Moreover, using the ROCK blocker Y-27632, we found an almost two fold enhancement of neurite length (Fig. 2). Most likely, the effects on neurite length of both compounds are mediated via their inhibitory action on the RhoA/ROCK pathway [18,25]. The pull down assay (Fig. 5) showed indeed that treatment of NT2 neurons with Ibuprofen decreased RhoA activation, while LPA, a stimulator of the Rho pathway increased activation. Both downstream ROCK blockers Y-27632 and Fasudil showed no effect.

The positive effects on neurite lengths after RhoA/ROCK inhibition are in line with several functional regeneration studies after spinal cord hemisection described in a systematic review. Pharmacological intervention causing blockage of RhoA/ROCK resulted in an overall increase to 15% in locomotor activity [26]. Human NT2 neurons have been reported as very susceptible to the RhoA activating chondroitin sulphate proteoglycans (CSPG) *in vitro* [15]. Grown on a CSPG substrate, they failed to grow neurites and neurite length was decreased to 20% of control. Blocking of RhoA with Fasudil prevented the CSPG mediated inhibitory effect at least partially [15]. Fasudil treatment rescued neurite lengths to 70% of control. In the present investigation, we cultured human 2wkRA neurons on a permissive substrate with concentrations of the analgetic Ibuprofen, which has also been discovered to block RhoA activation [18,25]. After 24 hours Ibuprofen treatment, neurite lengths were increased. Thus, we show to our knowledge for the first time an increase in neurite outgrowth of human neurons on a permissive substrate by RhoA/ROCK inhibiting agents (Figs. 1 and 2). Similar results after inhibition of RhoA have been found in SCI models of adult mice [14] retinal nerve, and thoracic spinal cord transection injury models of rats [25,27]. Subcutaneous administration of 60 mg/kg Ibuprofen per day resulted in rostral sprouting of corticospinal tract fibers [25]. Culturing rat pheochromocytoma neurons (PC12) with the RhoA activating agent LPA resulted in dramatically increased RhoA levels *in vitro* [25] Ibuprofen and indomethacin prevented this increase. Moreover, PC12 neurons exposed to myelin led to high levels of RhoA in cell lysates whereas treatment with Ibuprofen abolished this increase [25]. Since several studies suggested treatment with ROCK inhibitors as a promising therapy to treat spinal cord injuries [8,10,28,29], we tested whether the Rho kinase inhibitor Y-27632 could promote neurite outgrowth and elongation in similar manner as Ibuprofen does. Inhibition of Rho kinase, a downstream target of RhoA, indeed led to a dose-dependent increase (Fig. 2) of neurite lengths *in vitro*. This result confirmed the increased neurite elongation observed in neurons derived from mouse neural stem cells after Y-27632 treatment [13].

Increased RhoA levels after LPA treatment had no significant impact on long-term neurite outgrowth (Fig. 4). However, activation of RhoA by LPA resulted in a complete growth cone collapse and rapid neurite retraction within the time frame of 45 minutes (Fig. D, D’).

In comparison to earlier studies using human NT2 neurons [15], we demonstrated the neurite outgrowth enhancing effects of Ibuprofen and Y-27632 for human model neurons on a permissive substrate. Such findings might be important for regeneration in the peripheral nervous system, where axon outgrowth is not impaired by such a hostile environment as in the central nervous system [30,31].

Rather, for peripheral nervous system regeneration the time frame in which axons re-establish contact to distal nerve stumps is of more importance [32–34]. Within several days and weeks after transection or avulsion of ventral roots, extensive death of motor neurons of 50–80% occurred [35–38]. For humans, the gold standard for peripheral nerve repair is the transplantation of autologous scaffolds with moderate regeneration over nerve gaps not wider than 20 mm [39]. Levels of mRNA of RhoA were increased in injured rat DRG neurons [34]. Neurite lengths *in vitro* and an increase of number of axons *in vivo* were the results after application of the RhoA-ROCK inhibitor HA-1077 [34]. Here we show similar results in human model neurons as reported for rat DRG neurons [34] Application of Ibuprofen to PNS injuries may result in the bridging of longer nerve defects and a faster regeneration process with less or absent degeneration of nerve stumps.

Earlier studies in our laboratory have shown the effect of the cAMP pathway on neurite outgrowth in human NT2 neurons. Exogenously applied cAMP or treatment with the adenylyl cyclase activator, Forskolin, resulted in an increase of neurite extension to 150% of control [21]. Here we report that treatment with Ibuprofen increased lengths of neurites to a similar extent. This could be explained by earlier studies showing Rho inhibition via cAMP [40–42]. The cAMP-dependent kinase (PKA) phosphorylates RhoA, resulting in a translocation of membrane-bound RhoA to the cytosol and therefore a loss of function for RhoA.

### Inhibition of RhoA/ROCK induces neurite formation

Initiation and formation of neurites by severed neurons is a prerequisite for regeneration mechanisms [43]. Therefore we counted the number of neurites of human 2wkRA neurons cultured in RhoA-inhibiting Ibuprofen and ROCK-inhibiting Y-27632. Ibuprofen and Y-27632 incubation resulted in more neurons bearing neurites (Fig. 3). Inhibition of Rho kinases with Y-27632 resulted in a decrease of myristoylated alanine-rich C kinase substrate (MARCKS) in human SH-SY5Y cells [44]. Expression of an unphosphorylable mutant of MARCKS also led to an increase in neurite bearing cells. This effect could also be elicited by treatment with the insulin-like growth factor-1 (IGF-1) inducing a transient inactivation of RhoA [44]. In line with these results, neurite initiation was prevented after RhoA and ROCK activation [45]. Decreased RhoA activity on PC12 cells was observed after treatment with nerve growth factor (NGF), a major trigger for neuritogenesis of this cell line [46]. Treatment of CA1/CA3 hippocampal neurons of mice with Rho and ROCK inhibitors caused also neurite initiation [47].

In summary, here we showed for the first time that treatment of human neurons with a commercial pain reliever enhanced the neurite growth capacity. These encouraging in vitro results are in line with findings in experimental animals, showing that inhibition of RhoA-ROCK overcomes the regeneration inhibitory effects of myelin and chondroitin sulfate proteoglycans. Blocking of a single signal transduction pathway is rather unlikely to fully account for neurite regeneration. Since Ibuprofen additionally attenuates the inflammatory response caused by injury, therapeutic strategies [48,49] for the application of the drug close to the site of neural tissue damage should be explored.
